# Toad Glandular Secretions and Skin Extractions as Anti-Inflammatory and Anticancer Agents

**DOI:** 10.1155/2014/312684

**Published:** 2014-03-06

**Authors:** Ji Qi, C. K. Tan, Saeed M. Hashimi, Abu Hasanat Md. Zulfiker, David Good, Ming Q. Wei

**Affiliations:** ^1^Division of Molecular and Gene Therapies, Griffith Health Institute and School of Medical Science, Griffith University, Gold Coast, QLD 4222, Australia; ^2^School of Physiotherapy, Australian Catholic University, Banyo, QLD 4014, Australia

## Abstract

Toad glandular secretions and skin extractions contain many natural agents which may provide a unique resource for novel drug development. The dried secretion from the auricular and skin glands of Chinese toad (*Bufo bufo gargarizans*) is named Chansu, which has been used in Traditional Chinese Medicine (TCM) for treating infection and inflammation for hundreds of years. The sterilized hot water extraction of dried toad skin is named Huachansu (Cinobufacini) which was developed for treating hepatitis B virus (HBV) and several types of cancers. However, the mechanisms of action of Chansu, Huachansu, and their constituents within are not well reported. Existing studies have suggested that their anti-inflammation and anticancer potential were via targeting Nuclear Factor (NF)-*κ*B and its signalling pathways which are crucial hallmarks of inflammation and cancer in various experimental models. Here, we review some current studies of Chansu, Huachansu, and their compounds in terms of their use as both anti-inflammatory and anticancer agents. We also explored the potential use of toad glandular secretions and skin extractions as alternate resources for treating human cancers in combinational therapies.

## 1. Introduction

The link between inflammation and cancer was first suggested by Rudolf Virchow in 1863 [1], but this theory was largely ignored until recent decades [2]. Statistics have shown that up to 25% of cancer mortality worldwide is related to chronic inflammation [3]. Some pieces of the epidemiological evidence are detailed below.

- Infection with* Helicobacter pylori* is associated with gastric cancer and gastric mucosal lymphoma [4].
- Hepatitis B virus (HBV) and hepatitis C virus (HCV) related chronic infections contribute to increased risk of hepatocellular carcinoma (HCC) [5].
- Autoimmune diseases such as inflammatory bowel diseases (IBDs) may trigger colon cancer [6]. Similarly, prostatitis is associated with prostate cancer [7] and tobacco is also a promoter for lung carcinogenesis [8].
- Long-term use of nonsteroidal anti-inflammatory drugs (NSAIDs) reduces the risk of several cancers [9].

Recent studies have revealed the crosstalk between chronic inflammation and cancer [1, 10]. The microenvironment of tumours are typically rich in proinflammatory mediators, such as Tumour Necrosis Factor (TNF)-*α*, Interleukin (IL)-1, IL-6, IL-8, cyclooxygenases-2 (COX-2), and inducible nitrogen synthesis (iNOS) which are essential components of the tumour initiation and promotion [11, 12]. It has also been recognised that the transcription factor NF-*κ*B plays a central coordinator role between inflammation and cancer by regulating the above mentioned proinflammatory cytokines and enzymes [13]. Constitutive activation of NF-*κ*B signalling has been found in several types of tumours including breast, colon, prostate, skin, and lymphoid and mediates resistance to chemo- and radiotherapy [14]. Hence, therapeutic blockade of NF-*κ*B signalling has become a promising target for the prevention and treatment of cancer [15]. In another words, an agent that suppresses NF-*κ*B activation may have potential in the development of novel anticancer drugs [16].

Natural products have been used extensively in the treatment of many diseases [17]. Anti-inflammation and anticancer drugs developed from natural resources have drawn significant attention throughout the world [18]. The biodiversity of chemical compounds in the auricular and skin glands of toads makes them unique sources from which new therapeutic agents may be developed [19]. Additionally, toad glandular secretions have been used for treating infection and inflammation for centuries in Traditional Chinese Medicine (TCM) in China and East/Southern East Asian countries. Recent studies have indicated that toad glandular secretions and skin extractions can be both anti-inflammatory and anticancer agents. Therefore, this type of natural products may provide a potential new strategy as combinational and/or complimentary therapies for cancer treatment by targeting key NF-*κ*B signalling molecules and their pathways.

## 2. Inflammation and Cancer

Inflammation is part of a mechanism of innate immunity, providing protection from infection, injury, or irritation [20]. The common inflammatory symptoms include redness, heat, swelling, pain, and sometimes loss of function in the inflamed tissue [21]. Inflammation helps heal wounds by clearing the injurious agents and removing damaged tissue components [22]. However, if inflammation is not under control promptly, the inflammatory triggering factors continue stimulating the tissue; this could result in chronic inflammation, leading to a wide variety of diseases such as rheumatoid arthritis, heart attack, Alzheimer's disease, and cancer [23].

Cancer-related inflammation can be summarized into two categories. On one hand, chronic inflammatory conditions cause tissue damage which augment the risk of cancer development [24]. On the other hand, inflammation is also found in cancer tissues that arose without precancerous inflammation [25]. Genetic alterations which include mutations, activation of oncogenes, chromosomal rearrangement or amplification, and the inactivation of tumour-suppressor genes in tumour cells can induce an inflammatory microenvironment [26]. Regardless of the origin of the inflammatory condition, inflammatory microenvironment is essential for the maintenance and promotion of cancer progression [27].

## 3. NF-*κ*B and Its Regulated Gene Products in Inflammation and Cancer

**Figure 1:**
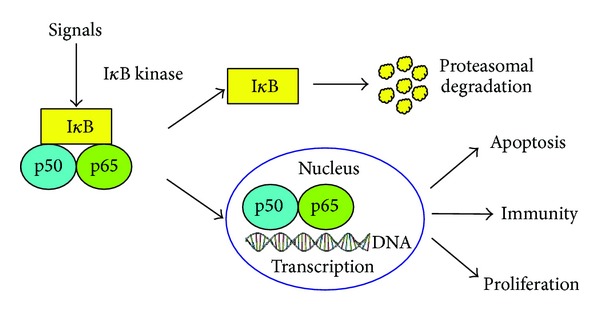
The activation process of NF-*κ*B.

In cancer-related inflammation, the expression of major proinflammatory gene products is regulated by NF-*κ*B which is an important transcription factor that is involved in many physiological processes including proliferation, apoptosis, tumourigenesis, inflammation, and various diseases including cancer [28]. There are five proteins of two classes in the mammalian NF-*κ*B family (Class 1: NF-*κ*B1/p50, NF-*κ*B2/p52; Class 2: RelA/p60, RelB, c-REL). In resting stage, NF-*κ*B is a heterodimer (e.g., p50 and p65) bound with Inhibitor *κ*B (I*κ*B) to remain in an inactive form in the cytoplasm. However, in response to a variety of stimuli, I*κ*B is phosphorylated by I*κ*B kinase, resulting in the translocation of NF-*κ*B into the nucleus to exert its function (Figure 1). The activation of NF-*κ*B is thought to be part of a stress response as it is activated by a variety of stimuli that include growth factors, cytokines, lymphokines, UV, pharmacological agents, and stress [10, 29]. The activation of NF-*κ*B also occurs in many malignant cells as a result of genetic mutation rather than in response to signals from surrounding cells [30]. Most carcinogens can activate NF-*κ*B, whereas in most tumour cells, it is kept as a constitutively active protein [31].

NF-*κ*B is considered the “master” gene transcription factor for promoting inflammation by regulating proinflammatory mediators. On the other hand, NF-*κ*B itself is activated by these same inflammatory cytokines [32, 33]. Studies in mouse models show that NF-*κ*B and TNF-*α* are linked with cancer progression [34, 35]. TNF-*α* produced by neighbouring inflammatory cells can control the activation of NF-*κ*B and its localisation in cells; this has also been reported by Greten et al., when they were studying a mouse colitis-associated cancer model [35]. Their work does not directly implicate TNF-*α*, though, but instead found enhanced production of several proinflammatory cytokines, including TNF-*α*, in the tumour microenvironment during the development of cancer. Both reports demonstrate that the reduction in NF-*κ*B activation leads to lower incidence of cancer development [34, 35].

Studies have also shown that other proinflammatory molecules are indispensable for the initiation, promotion, and progression of tumour [36–39]. Among these proinflammatory mediators, the crucial roles of TNF-*α*, IL-1, IL-6, IL-8, COX-2, and iNOS have been mostly highlighted [40, 41]. It has been shown that the expressions of all these mediators are regulated by transcription factor NF-*κ*B [35, 36, 42]. The identification of these factors has provided the molecular basis for the role of inflammation in cancer [23]. They have also served as the molecular targets in drug design strategies for both cancer prevention and therapy [23]. We will detail some of these proinflammatory factors in the latter part of this review.

NF-*κ*B dimer (e.g., p50 and p65) binds with I*κ*B to remain an inactive condition in resting cells. For responding to the stimuli, I*κ*B is degraded by proteasome and the free NF-*κ*B dimer is translocated from cytoplasm to nucleus. The activation of NF-*κ*B controls many physiological processes such as apoptosis, immunity, and proliferation.

## 4. Current Study of Anti-Inflammatory Agents in Cancer Prevention and Treatment

Currently, many anticancer agents have been used for the treatment of inflammatory diseases, such as rheumatoid arthritis. Meanwhile, the eliminating of inflammation may provide an efficiency approach for cancer prevention and therapy [43]. As we mentioned existing epidemiological data suggest that NSAIDs, the most common of which are aspirin or ibuprofen, naproxen, and indomethacin, vastly lower the incidence and motility of breast, colorectal, and lung cancer [44]. NSAIDs are known as potent anti-inflammatory agents that act through the inhibition of the COX enzyme and the subsequent inhibition of prostaglandins which are catalysed by COX enzymes at the site of inflammation [45]. This type of drug is usually accompanied by side effects; however, some studies have suggested that selective COX-2 inhibitors such as celecoxib may produce superior anti-inflammatory drugs with substantial safety advantages over existing NSAIDs [46].

Many other cytokine inhibitors are also undergoing preclinical and clinical trials for the treatment of cancer. The therapeutic anti-IL-6 antibody, siltuximab (CNTO328), has been evaluated in Phase II trials of castration-resistant prostate cancer [47, 48]. Moreover, the anti-TNF-*α* antibody has shown a significant therapeutic effect in inflammatory and autoimmune diseases which indicates that they may also have potential in the use for cancer therapy [49–51].

## 5. The Use of Chansu and Huachansu in Traditional Chinese Medicine

The dried secretions of the auricular and skin glands of Bufo* bufo gargarizans Cantor* or B.* melanostictus Schneider *is known as Chansu in TCM [52]. It can be used as a single agent or more commonly in combination with other TCMs in a recipe, for example, Liu-shen-wan [43]. Chansu has been widely used for hundreds of years as an anodyne, cardiotonic, antimicrobial, and local anaesthetic agent [53]. The anti-infection and anti-inflammatory effect of Chansu has been recorded in many observational researches in Chinese publications and medical classics for the treatment of otitis media, periodontitis, hepatitis, and arthritis [54].

Toad glandular secretions and skin extractions can be made to different types including oral solution, ointment, injection, and coating agent. One of the most widely used commercial preparation is Huachansu (Cinobufacini), which is a sterilized hot water extract of dried toad skin [55]. Since 1991, Cinobufacini has been officially approved by the Chinese Food and Drug Administration as a regimen for treating patients with HBV and several types of cancer including liver, lung, colon, and pancreatic cancer [56].

An* in vivo* assay showed that the Cinobufacini injection inhibits the growing of mouse Lewis lung cancer cells with the response rate of 45%~50% and prolongs their life [57]. Clinical trials of Cinobufacini injection have been conducted since the 1970s within China. The results have demonstrated the anticancer effect of Cinobufacini injection in advanced hepatocellular carcinoma and lung cancer patients with a total response rate of 10% and 16%, respectively [58]. A Phase I clinical trial conducted by Meng et al. further examined the tolerable toxicity in patients. The result showed that Cinobufacini injection can be tolerated up to 8 times higher than normal administrated does. Up to 40% lung and liver cancer patients had tumours stabilised in this trail [59]. Additionally, Cinobufacini injection promoted the efficacy of the conventional therapies while lowering their toxicity when it was used in combination with chemotherapy or radiotherapy [60, 61]. To fully reveal the anticancer property as well as the underlined mechanism of this drug, more preclinical study and clinical trials are needed.

## 6. Compounds in Chansu and Huachansu

Several classes of compounds including bufadienolides, steroids, indole alkaloids, peptides, organic acids, and others have been analysed and isolated by various methods from the auricular and skin glands of different species of toads as listed in Table 1 [62, 63].

**Figure 2:**
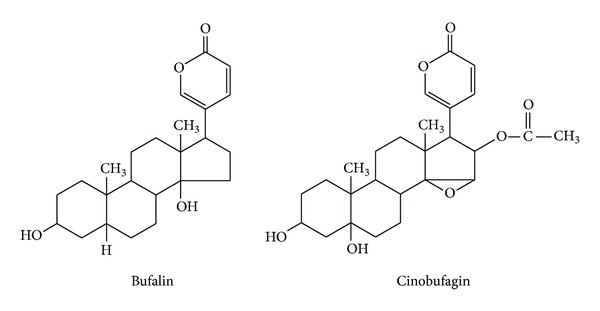
Chemical structure of bufalin and cinobufagin.

Bufadienolides such as bufalin and cinobufagin (Figure 2) are recognised as the major bioactive components in Chansu and Huachansu. This group of chemicals has been known as cardiac glycosides (Table 1) [56, 64].

## 7. The Anticancer Studies of Cardiac Glycosides

Therapeutic uses of cardiac glycosides primarily are involved in the treatment of cardiac failure. However, recent studies showed that this type of chemical structure also exerts effects in cancer therapy [56, 64]. Ye et al. showed bufalin and other cardiac glycoside inhibitors of the sodium pump block the activation of the transcription factors IRF3 and NF-*κ*B by inhibiting the induction of interferon-*β*. They have also showed that bufalin blocks tumour necrosis factor (TNF) signalling, at least in part by mediating the nuclear translocation of NF-*κ*B. [65]. Yang et al. showed that cardiac glycosides block the activation of the TNF-*α*/NF-*κ*B signalling pathway in Hela cells [66]. Another study showed that cardiac glycosides prevent lipopolysaccharide (LPS)-induced activation of proinflammatory cytokines including TNF-*α*, IL-1 and IL-6 in whole blood at least partly through an NF-*κ*B-dependent mechanism and also can inhibit IL-8 in various cells types [67]. Moreover, a mouse model study also showed that this group of cardiac glycoside structurally related compounds can suppress the expression of NF-*κ*B and I*κ*B as well as COX-2 in skin cancer [68]. These findings indicate that cardiac glycosides including bufalin have a potent role in treating inflammatory and autoimmune diseases.

## 8. Mechanisms of Action of Toad Glandular Secretions and Skin Extractions as Anti-Inflammatory and Anticancer Agents

### 8.1. Targeting Cancer-Related Inflammation

The molecular mechanisms of toad glandular secretions and skin extractions have recently been the focus of several studies and the anti-inflammatory property has been looked at in several inflammatory and cancer models (Table 2). However, current studies are limited and further investigations are required to fill the gaps. Here, we review some current research progresses.

Dong et al. investigated the effect of the single compound cinobufagin on liver cancer cell line HepG2 and found that cinobufagin significantly suppressed NF-*κ*B p65 protein expression [74]. Jiang et al. found that bufalin at the concentration of 2.5–10 *μ*M reduced expression level of COX-2 protein in A549 cells. In addition, they also found that bufalin suppressed the phosphorylation and expression of NF-*κ*B [75]. Chen et al. demonstrated that bufalin inhibits migration and invasion in human hepatocellular carcinoma SK-Hep1 cells at least partly by the inhibition of NF-*κ*B signalling pathway [76]. In another study, the nitric oxide (NO) regulatory effect of Chansu and bufalin was studied by Bhuiyan et al. on BeWo cells. Their study indicated that Chansu at the concentration of 5–10 *μ*g/mL significantly up-regulated NO production to 110% of basal control value, while at a higher concentration of 40–80 *μ*g/mL, Chansu decreased NO production. A similar result was also observed with bufalin in this study [77]. INOS is one of three identified enzymes of nitric oxide synthase family, which catalyse the production of nitric oxide (NO) from L-arginine [82]. iNOS-derived NO is a vital cellular signalling molecule which is associated with many physiological and pathological processes, including inflammation and carcinogenesis [83]. Increased expression of iNOS has been observed in tumours as well as in chronic inflammatory diseases [84]. Chronic inflammatory conditions induce overexpression of iNOS which generate sustainable NO, and its reactive intermediates are mutagenic, which result in DNA damage or absence of DNA repair. Recent studies indicate NO as a key signalling molecule that regulates processes of tumourigenesis. The development of selective inhibitors of iNOS and NO-releasing agents is underway and this may lead to important strategies for chemoprevention of cancer [85].

In another study, Kim et al. found that Chansu significantly inhibits iNOS and COX-2 mRNA and protein expression as well as the production of NO and Prostaglandin E2 (PGE2) in LPS-stimulated BV2 microglial cells. Their study also showed that Chansu inhibited the production of TNF-*α*, IL-1*β*, and IL-12 and the degradation of I*κ*B-*α*, which was considered to be inhibitor of NF-*κ*B [78]. Ko et al. indicated that Chansu inhibited the level of COX-2 mRNA and protein expression and also the PGE2 synthesis without significant influence on the level of COX-1 in human bladder carcinoma T24 cells [79].

In an* in vitro* study on tumour cells, Wang et al. investigated the effects of Huachansu on NF-*κ*B, COX-2 and the proinflammatory cytokines IL-6 and IL-8 induced by TNF-*α* in the A549 cell line. They found that Huachansu inhibited NF-*κ*B and COX-2 activation induced by TNF-*α* and decreased the expression levels of proinflammatory cytokines IL-6 and IL-8 [80].

Chansu can be also combined with other TCMs in a recipe, such as Liu-shen-wan (LSW), for the treatment of many other health conditions. The ingredients of LSW are Zhenzhufeng, Niuhuang, Shexiang, Xionghuang, Bingpian, and Chansu [86]. It has been used for various purulent infections clinically because of immune-enhancing, antimicrobial, anti-inflammatory, and cardiotonic activities. Ma et al. showed that LSW significantly reduced the levels of circulating TNF-*α* while there was a slight decrease in the levels of IL-1 in mouse macrophage, which suggests that LSW enhances the beneficial host defense function at the primary site of infection and weakens harmful inflammatory response at other sites [81].

### 8.2. Anti-Infection

The antimicrobial activity of telocinobufagin and marinobufagin was performed by liquid growth inhibition against two types of bacteria. The result has shown that the minimum inhibitory concentrations of telocinobufagin and marinobufagin were 64.0 and 16.0 *μ*g/mL, respectively, for* E. coli* while both 128 *μ*g/mL for* S. aureus *[87]. Cui et al. examined the antihepatitis B virus (HBV) activities of Cinobufacini and its active component bufalin and cinobufagin in the human HBV-transfected cell line HepG2.2.15. Results suggested that Cinobufacini had more potent activity against HBV antigen secretion than its components bufalin and cinobufagin and this inhibitory role was attributed to the specific inhibition of HBV mRNA expression [88].

### 8.3. Immunomodulatory Effect

Numerous diseases including cancer are often associated with depression of the immune system [89]. Some researchers have also shown that Chansu and Huachansu, as well as their active components, are immunomodulators which enhance the function of immune system.

In an* in vitro* assay, cinobufagin was shown to have markedly effects on the cell proliferation splenocytes and peritoneal macrophages (PMF) and enhanced the phagocytic activation of PMF. Additionally, the ratios of CD4+ CD8+ double-positive T-cell and S-phase cells of splenic lymphocytes are also increased by the treatment of cinobufagin. Furthermore, the levels of Th1 cytokines, including interferon-*γ* and TNF-*α*, were significantly enhanced, whereas the levels of the Th2 cytokine IL-4 and IL-10 were dramatically reduced. As a result, the percentage of Th1/Th2 is also up-regulated [90]. In a study by Cao et al., telocinobufagin was found to significantly stimulate splenocyte proliferation and to enhance natural killer cell and PMF activation in BALB/c mice. Telocinobufagin significantly increased the percentage of CD4+ and CD8+ T cell populations. Moreover, Cao et al. found that the levels of Th1 cytokines including IL-2, IL-12, IFN-*γ* and TNF-*α* were sharply elevated after telocinobufagin treatment, while the level of the Th2 cytokine IL-4 was significantly lowered, leading to an increased ratio of Th1/Th2 [91]. Liu et al. found that Cinobufacini injection at the concentration of 0.0625–0.5 *μ*g/mL increased the expression of IL-2 in splenic lymphocytes of mice with enhanced T lymphocyte dependent immune responses [92]. These results suggest that the active compounds in Chansu and Huachansu have potential immunomodulatory properties. More work is needed for development of novel immunotherapeutic agents from toad glandular secretions and skin extractions for treating cancer.

### 8.4. Induction of Tumour Cells into Apoptosis

In recent years, there are a growing number of studies indicating that apoptosis is a key process in cancer development and progression. Apoptosis is a specific process that leads to programmed cell death, which is essential in the homeostasis of normal tissues of the body and the dysregulation of this process may cause many diseases such as cancer.

The expression of tumour cell apoptosis-related genes plays an important regulatory role in the tumour development process. Some gene deletion, mutation, or overexpression within this process may lead to uncontrolled cell proliferation or apoptosis blockage. Therefore, regulation of apoptosis-related genes or their corresponding products improves the sensitivity of tumour cells to drugs and induces cells apoptosis which is the key in cancer treatment.

A number of studies have revealed the effect of Chansu, Huachansu, and their single compound on inducing lung, colon, breast, and prostate cancer and a wide range of tumour cell lines into apoptosis [93]. Yun et al. have indicated that Chansu induces the apoptosis of A549 cells through a signalling cascade of death receptor-mediated extrinsic and mitochondria-mediated intrinsic caspase pathways. In their study, the treatment with Chansu down-regulated the antiapoptotic Bcl-2 expression and up-regulated proapoptotic Fas ligand and death receptor 4 while reduced the mitochondrial membrane potential. Additionally, the proteolytic activation of caspases and a concomitant degradation of poly- (ADP-ribose-) polymerase and *β*-catenin protein were found after treatment with Chansu. Cleavage of Bid and a downregulation of the inhibitor of apoptosis family of proteins were also observed in this study [94]. Another study from Wang et al. concluded that the apoptosis of A549 cells can be induced by Cinobufacini through the decreased expression of survivin and the increased caspase-3 activity [95]. In a study of bufadienolides, the antiproliferative and apoptotic mechanisms of prostate cancer cells LNCaP, DU145, and PC3 treated with bufalin and cinobufagin were investigated. After treatment with bufalin and cinobufagin, the caspase-3 activity, protein expression of caspase-3, -8, and -9, and other apoptotic modulators including mitochondrial Bax and cytosolic cytochrome *c* were elevated. The increased expression of p53 was only observed in LNCaP cells. Additionally, the reduction of p53 by antisense* TP53* restored the cell viability suppressed by bufalin and cinobufagin. Their study also indicated that the increased expression of Fas was more significant in DU145 and PC3 cells with mutant p53 than in LNCaP cells. The transfection of Fas small interfering RNA restored cell viability in the bufadienolide-treated cells [96, 97].

### 8.5. Efficacy and Safety

The side effects caused by Chansu and Huachansu are thought to be mainly contributed by its cardiac glycoside property [98]. However, a recent study showed that it is possible to alleviate such toxicity by using long circulating liposomes which could enhance the therapeutic effects of bufadienolides and reduce their toxicity. Hu et al. found that the bufadienolide liposomes had an LD50 that was 3.5 time higher than the LD50 of bufadienolide solution without severe toxicity [99]. Another study also showed that bufadienolide-loaded nanostructured lipid carriers were safe when given by intravenous injection with reduced toxicity [100]. Ma et al. showed that Bezoar Bovis, which is a TCM usually in combination with Chansu, can protect against Chansu-induced acute toxicity in mice. Additionally, their anti-inflammatory study also showed that Bezoar Bovis did not reduce the anti-inflammatory activity of Chansu and Huachansu on concanavalin-A-stimulated proliferation of human peripheral blood mononuclear cells [101].

## 9. Conclusion

Current conventional cancer therapies are mostly surgery, chemotherapy, and radiotherapy. However, problems such as metastasis and tumour resistance to chemotherapy and radiotherapy have seriously limited the therapeutic effects of existing clinical treatments [102]. Studies have shown that toad glandular secretions and skin extractions contain many chemical compounds which possess both anti-inflammatory and anticancer properties and can be used as complementary and combinational agents to conventional therapies on cancer treatment. Thus, it is invaluable to study the anti-inflammatory activity of their bioactive constitutes by targeting inflammatory mediators in cancer not only to establish anti-inflammatory mechanisms, but also to develop a new class of anti-inflammatory agents which may be useful in the prevention and treatment of cancer. Present studies have shown that toad medicines decrease inflammation and cancer through a variety of mechanisms, including inhibition of NF-*κ*B and its signalling molecules and pathways. Preclinical studies also suggested that many cardiac glycosides derived from Chansu and Huachansu have potent activity against cancer cells, which prevent carcinogenesis or metastasis of existing tumours. Therefore, toad glandular secretion and skin extraction have a real potential as resources for the development of therapeutical agents for preventing or treating human cancers by inducing apoptosis, sensitizing cancer cells to conventional cancer therapies, or protecting host cells from any side effects.

## Figures and Tables

**Table 1:** Chemical properties of Chansu and Huachansu.

Objective	Method	Compounds	Reference
Chansu	HPLC and LC-DAD-MS/MS	gamabufotalin arenobufagin telocinobufagin bufotalin cinobufotalin bufalin cinobufagin resibufogenin	[69]

Chansu	High-speed counter-current chromatography	arenobufagin telocinobufagin bufotalin cinobufotalin bufalin resibufogenin cinobufagin	[70]

Chansu	HPLC/APCI-MS/MS	bufalin arenobufagin bufotalin telocinobufagin *ψ*-Bufarenogin bufotalinin cinobufotalin gamabufotalin	[71]

Huachansu	HPLC	bufalin, cinobufagin resibufogenin	[72]

Huachansu	HPLC-QqQ MS	bufalin cinobufagin recinobufagin cinobufotalin telocinobufagin, gamabufotalin arenobufagin, bufotalin	[73]

**Table 2:** Targets of toad medicines in the treatment of cancer related inflammation.

Drugs	Targets	References
Cinobufagin	NF-*κ*B	[74]

Bufalin	NF-*κ*B, COX-2	[75]
	NF-*κ*B	[76]
	NO	[77]

Chansu	NO	[77]
	iNOS, COX-2, NO, PGE2, TNF-*α*, IL-1*β*, IL-12, I*κ*B-*α*	[78]
	COX-2, PGE2	[79]

Huachansu	NF-*κ*B, COX-2, IL-6, IL-8	[80]

Liu-shen-wan	TNF-*α*, IL-1	[81]

## References

[1] Balkwill F, Mantovani A (2001). Inflammation and cancer: back to virchow?. *The Lancet*.

[2] Colotta F, Allavena P, Sica A, Garlanda C, Mantovani A (2009). Cancer-related inflammation, the seventh hallmark of cancer: links to genetic instability. *Carcinogenesis*.

[3] Hagemann T, Balkwill F, Lawrence T (2007). Inflammation and cancer: a double-edged sword. *Cancer Cell*.

[4] Uemura N, Okamoto S, Yamamoto S (2001). *Helicobacter pylori* infection and the development of gastric cancer. *The New England Journal of Medicine*.

[5] Naugler WE, Sakurai T, Kim S (2007). Gender disparity in liver cancer due to sex differences in MyD88-dependent IL-6 production. *Science*.

[6] Jess T, Loftus EV, Velayos FS (2006). Risk of intestinal cancer in inflammatory bowel disease: A Population-Based Study from Olmsted County, Minnesota. *Gastroenterology*.

[7] De Marzo AM, Platz EA, Sutcliffe S (2007). Inflammation in prostate carcinogenesis. *Nature Reviews Cancer*.

[8] Multhoff G, Molls M, Radons J (2012). Chronic inflammation in cancer development. *Frontiers in Immunology*.

[9] Gupta RA, DuBois RN (2001). Colorectal cancer prevention and treatment by inhibition of cyclooxygenase-2. *Nature Reviews Cancer*.

[10] Coussens LM, Werb Z (2002). Inflammation and cancer. *Nature*.

[11] Burke F, Relf M, Negus R, Balkwill F (1996). A cytokine profile of normal and malignant ovary. *Cytokine*.

[12] Mantovani A, Garlanda C, Allavena P (2010). Molecular pathways and targets in cancer-related inflammation. *Annals of Medicine*.

[13] Karin M, Greten FR (2005). NF-*κ*B: linking inflammation and immunity to cancer development and progression. *Nature Reviews Immunology*.

[14] Lee CH, Jeon Y-T, Kim S-H, Song Y-S (2007). NF-*κ*B as a potential molecular target for cancer therapy. *BioFactors*.

[15] Luqman S, Pezzuto JM (2010). NF*κ*B: a promising target for natural products in cancer chemoprevention. *Phytotherapy Research*.

[16] Yamamoto Y, Gaynor RB (2001). Therapeutic potential of inhibition of the NF-*κ*B pathway in the treatment of inflammation and cancer. *Journal of Clinical Investigation*.

[17] Da Rocha AB, Lopes RM, Schwartsmann G (2001). Natural products in anticancer therapy. *Current Opinion in Pharmacology*.

[18] Yadav VR, Prasad S, Sung B, Kannappan R, Aggarwal BB (2010). Targeting inflammatory pathways by triterpenoids for prevention and treatment of cancer. *Toxins*.

[19] Clarke BT (1997). The natural history of amphibian skin secretions, their normal functioning and potential medical applications. *Biological Reviews of the Cambridge Philosophical Society*.

[20] Weiss U (2008). Inflammation. *Nature*.

[21] Mariotti A (2004). A primer on inflammation. *Compendium of Continuing Education in Dentistry*.

[22] Hart J (2002). Inflammation. 1: its role in the healing of acute wounds. *Journal of Wound Care*.

[23] Mantovani A, Allavena P, Sica A, Balkwill F (2008). Cancer-related inflammation. *Nature*.

[24] Balkwill F, Coussens LM (2004). Cancer: an inflammatory link. *Nature*.

[25] Mantovani A, Garlanda C, Allavena P (2010). Molecular pathways and targets in cancer-related inflammation. *Annals of Medicine*.

[26] Borrello MG, Alberti L, Fischer A (2005). Induction of a proinflammatory program in normal human thyrocytes by the RET/PTC1 oncogene. *Proceedings of the National Academy of Sciences of the United States of America*.

[27] Aggarwal BB, Shishodia S, Sandur SK, Pandey MK, Sethi G (2006). Inflammation and cancer: how hot is the link?. *Biochemical Pharmacology*.

[28] Aggarwal BB, Vijayalekshmi RV, Sung B (2009). Targeting inflammatory pathways for prevention and therapy of cancer: short-term friend, long-term foe. *Clinical Cancer Research*.

[29] Luo J-L, Kamata H, Karin M (2005). IKK/NF-*κ*B signaling: balancing life and death—a new approach to cancer therapy. *Journal of Clinical Investigation*.

[30] Baud V, Karin M (2009). Is NF-*κ*B a good target for cancer therapy? Hopes and pitfalls. *Nature Reviews Drug Discovery*.

[31] Karin M (2006). Nuclear factor-*κ*B in cancer development and progression. *Nature*.

[32] Yu YY, Li Q, Zhu ZG (2005). NF-*κ*B as a molecular target in adjuvant therapy of gastrointestinal carcinomas. *European Journal of Surgical Oncology*.

[33] Yang G, Yu F, Fu H (2007). Identification of the distinct promoters for the two transcripts of apoptosis related protein 3 and their transcriptional regulation by NFAT and NF*κ*B. *Molecular and Cellular Biochemistry*.

[34] Pikarsky E, Porat RM, Stein I (2004). NF-*κ*B functions as a tumour promoter in inflammation-associated cancer. *Nature*.

[35] Greten FR, Eckmann L, Greten TF (2004). IKK*β* links inflammation and tumorigenesis in a mouse model of colitis-associated cancer. *Cell*.

[36] Grivennikov SI, Greten FR, Karin M (2010). Immunity, inflammation, and cancer. *Cell*.

[37] Moore RJ, Owens DM, Stamp G (1999). Mice deficient in tumour necrosis factor-alpha are resistant to skin carcinogenesis. *Nature Medicine*.

[38] Voronov E, Shouval DS, Krelin Y (2003). IL-1 is required for tumour invasiveness and angiogenesis. *Proceedings of the National Academy of Sciences of the United States of America*.

[39] Enzler T, Gillessen S, Manis JP (2003). Deficiencies of GM-CSF and interferon *γ* link inflammation and cancer. *Journal of Experimental Medicine*.

[40] Didonato JA, Mercurio F, Karin M (2012). NF-*κ*B and the link between inflammation and cancer. *Immunological Reviews*.

[41] Dranoff G (2004). Cytokines in cancer pathogenesis and cancer therapy. *Nature Reviews Cancer*.

[42] Rakoff-Nahoum S, Paglino J, Eslami-Varzaneh F, Edberg S, Medzhitov R (2004). Recognition of commensal microflora by toll-like receptors is required for intestinal homeostasis. *Cell*.

[43] Rayburn ER, Ezell SJ, Zhang R (2009). Anti-inflammatory agents for cancer therapy. *Molecular and Cellular Pharmacology*.

[44] Langman MJS, Cheng KK, Gilman EA, Lancashire RJ (2000). Effect of anti-inflammatory drugs on overall risk of common cancer: case-control study in general practice research database. *British Medical Journal*.

[45] Cuzick J, Otto F, Baron JA (2009). Aspirin and non-steroidal anti-inflammatory drugs for cancer prevention: an international consensus statement. *The Lancet Oncology*.

[46] Khan Z, Khan N, Tiwari RP, Sah NK, Prasad GBKS, Bisen PS (2011). Biology of Cox-2: an application in cancer therapeutics. *Current Drug Targets*.

[47] Dorff TB, Goldman B, Pinski JK (2010). Clinical and correlative results of SWOG S0354: a phase II trial of CNTO328 (siltuximab), a monoclonal antibody against interleukin-6, in chemotherapy-pretreated patients with castration-resistant prostate cancer. *Clinical Cancer Research*.

[48] Fizazi K, De Bono JS, Flechon A (2012). Randomised phase II study of siltuximab (CNTO 328), an anti-IL-6 monoclonal antibody, in combination with mitoxantrone/prednisone versus mitoxantrone/prednisone alone in metastatic castration-resistant prostate cancer. *European Journal of Cancer*.

[49] Feldmann M (2002). Development of anti-TNF therapy for rheumatoid arthritis. *Nature Reviews Immunology*.

[50] Feldmann M, Maini RN (2003). TNF defined as a therapeutic target for rheumatoid arthritis and other autoimmune diseases. *Nature Medicine*.

[51] Sandborn WJ, Hanauer SB (1999). Antitumor necrosis factor therapy for Inflammatory Bowel Disease: a review of agents, pharmacology, clinical results, and safety. *Inflammatory Bowel Diseases*.

[52] Qi F, Li A, Inagaki Y (2011). Antitumor activity of extracts and compounds from the skin of the toad Bufo *bufo gargarizans* Cantor. *International Immunopharmacology*.

[53] Qi F, Li A, Inagaki Y (2010). Chinese herbal medicines as adjuvant treatment during chemo- or radio-therapy for cancer. *Bioscience trends*.

[54] Chen K, Jensen H (1929). Armacognostic study of Ch’an Su, the dried venom of the Chinese toad. *Journal of the American Pharmaceutical Association*.

[55] Gomes A, Bhattacharjee P, Mishra R (2010). Anticancer potential of animal venoms and toxins. *Indian Journal of Experimental Biology*.

[56] Wang L, Raju U, Milas L (2011). Huachansu, containing cardiac glycosides, enhances radiosensitivity of human lung cancer cells. *Anticancer Research*.

[57] Wu WY, Chai XS, Liu WS (2004). Synergistic anti-cancer effects and mechanisms of huachansu plus vinorelbine on Lewis lung cancer cell in mice. *China Oncology*.

[58] Cui Y, Zuo X, Qin S (2002). Clinical trial of Huachansu injection for anti-cancer efficacy. *Jiangsu Journal of Clinical Medicine*.

[59] Meng Z, Yang P, Shen Y (2009). Pilot study of huachansu in patients with hepatocellular carcinoma, nonsmall-cell lung cancer, or pancreatic cancer. *Cancer*.

[60] Xie X, Huang X, Li J (2013). Efficacy and safety of Huachansu combined with chemotherapy in advanced gastric cancer: a meta-analysis. *Medical Hypotheses*.

[61] Wang L, Raju U, Milas L (2011). Huachansu, containing cardiac glycosides, enhances radiosensitivity of human lung cancer cells. *Anticancer Research*.

[62] Wang D-L, Qi F-H, Tang W, Wang F-S (2011). Chemical constituents and bioactivities of the skin of Bufo *bufo gargarizans* cantor. *Chemistry and Biodiversity*.

[63] Gao H, Zehl M, Leitner A, Wu X, Wang Z, Kopp B (2010). Comparison of toad venoms from different Bufo species by HPLC and LC-DAD-MS/MS. *Journal of Ethnopharmacology*.

[64] Newman RA, Yang P, Pawlus AD, Block KI (2008). Cardiac glycosides as novel cancer therapeutic agents. *Molecular Interventions*.

[65] Ye J, Chen S, Maniatis T (2011). Cardiac glycosides are potent inhibitors of interferon-*β* gene expression. *Nature Chemical Biology*.

[66] Yang Q, Huang W, Jozwik C (2005). Cardiac glycosides inhibit TNF-*α*/NF-*κ*B signaling by blocking recruitment of TNF receptor-associated death domain to the TNF receptor. *Proceedings of the National Academy of Sciences of the United States of America*.

[67] Manna SK, Sreenivasan Y, Sarkar A (2006). Cardiac glycoside inhibits IL-8-induced biological responses by downregulating IL-8 receptors through altering membrane fluidity. *Journal of Cellular Physiology*.

[68] Afaq F, Saleem M, Aziz MH, Mukhtar H (2004). Inhibition of 12-O-tetradecanoylphorbol-13-acetate-induced tumor promotion markers in CD-1 mouse skin by oleandrin. *Toxicology and Applied Pharmacology*.

[69] Gao H, Zehl M, Leitner A, Wu X, Wang Z, Kopp B (2010). Comparison of toad venoms from different Bufo species by HPLC and LC-DAD-MS/MS. *Journal of Ethnopharmacology*.

[70] Li J, Ma X, Li F (2010). Preparative separation and purification of bufadienolides from Chinese traditional medicine of ChanSu using high-speed counter-current chromatography. *Journal of Separation Science*.

[71] Ye M, Guo H, Guo H, Han J, Guo D (2006). Simultaneous determination of cytotoxic bufadienolides in the Chinese medicine ChanSu by high-performance liquid chromatography coupled with photodiode array and mass spectrometry detections. *Journal of Chromatography B*.

[72] Su YH, Huang XQ, Zhang DZ (2003). HPLC separation and determination of bufadienolide in cinobufacini injection. * Chinese Traditional Patent Medicine*.

[73] Wu X, Zhao H, Wang H (2012). Simultaneous determination of eight bufadienolides in cinobufacini injection by HPLC coupled with triple quadrupole mass spectrometry. *Journal of Separation Science*.

[74] Dong Y-Q, Ma W-L, Gu J-B, Zheng W-L (2010). Effect of cinobufagin on nuclear factor-kappaB pathway in HepG2 cells. *Nan Fang Yi Ke Da Xue Xue Bao*.

[75] Jiang Y, Zhang Y, Luan J (2010). Effects of bufalin on the proliferation of human lung cancer cells and its molecular mechanisms of action. *Cytotechnology*.

[76] Chen YY, Lu HF, Hsu SC (2013). Bufalin inhibits migration and invasion in human hepatocellular carcinoma SK-Hep1 cells through the inhibitions of NF-kB and matrix metalloproteinase-2/-9-signaling pathways. *Environmental Toxicology*.

[77] Bhuiyan MBA, Fant ME, Dasgupta A (2003). Study on mechanism of action of Chinese medicine Chan Su: dose-dependent biphasic production of nitric oxide in trophoblastic BeWo cells. *Clinica Chimica Acta*.

[78] Kim MH, Lyu JH, Lyu SA (2008). Inhibitory effect of Chan-Su on the secretion of PGE2 and NO in LPS-stimulated BV2 microglial cells. *Korean Journal of Oriental Physiology and Pathology*.

[79] Ko WS, Park TY, Park C (2005). Induction of apoptosis by Chan Su, a traditional Chinese medicine, in human bladder carcinoma T24 cells. *Oncology reports*.

[80] Wang J-Y, Chen L, Zheng Z, Wang Q, Guo J, Xu L (2012). Cinobufocini inhibits NF-*κ*B and COX-2 activation induced by TNF-*α* in lung adenocarcinoma cells. *Oncology Reports*.

[81] Ma H, Kou J, Zhu D, Yan Y, Yu B (2006). Liu-Shen-Wan, a traditional Chinese medicine, improves survival in sepsis induced by cecal ligation and puncture via reducing TNF-*α* levels, MDA content and enhancing macrophage phagocytosis. *International Immunopharmacology*.

[82] Andrew PJ, Mayer B (1999). Enzymatic function of nitric oxide synthases. *Cardiovascular Research*.

[83] Korhonen R, Lahti A, Kankaanranta H, Moilanen E (2005). Nitric oxide production and signaling in inflammation. *Current Drug Targets*.

[84] Singh S, Gupta AK (2011). Nitric oxide: role in tumour biology and iNOS/NO-based anticancer therapies. *Cancer Chemotherapy and Pharmacology*.

[85] Lala PK, Chakraborty C (2001). Role of nitric oxide in carcinogenesis and tumour progression. *Lancet Oncology*.

[86] Hong Z, Chan K, Yeung HW (1992). Simultaneous determination of bufadienolides in the traditional Chinese medicine preparation, Liu-Shen-Wan, by liquid chromatography. *Journal of Pharmacy and Pharmacology*.

[87] Cunha Filho GA, Schwartz CA, Resck IS (2005). Antimicrobial activity of the bufadienolides marinobufagin and telocinobufagin isolated as major components from skin secretion of the toad Bufo rubescens. *Toxicon*.

[88] Cui X, Inagaki Y, Xu H (2010). Anti-hepatitis B virus activities of cinobufacini and its active components bufalin and cinobufagin in HepG2.2.15 Cells. *Biological and Pharmaceutical Bulletin*.

[89] Ehrke MJ (2003). Immunomodulation in cancer therapeutics. *International Immunopharmacology*.

[90] Wang X-L, Zhao G-H, Zhang J (2011). Immunomodulatory effects of cinobufagin isolated from Chan Su on activation and cytokines secretion of immunocyte in vitro. *Journal of Asian Natural Products Research*.

[91] Cao Y, Song Y, An N (2009). The effects of telocinobufagin isolated from Chan Su on the activation and cytokine secretion of immunocytes in vitro. *Fundamental and Clinical Pharmacology*.

[92] Liu X, Liu K, Yang Y (2005). Effects of cinobufacini on the growth of Hela cell and IL-2 production of murine spleen lymphocytes. *The Journal of Immunology*.

[93] Li C, Hashimi SM, Cao S (2013). The mechanisms of chansu in inducing efficient apoptosis in colon cancer cells. *Evidence-Based Complementary and Alternative Medicine*.

[94] Yun HR, Yoo HS, Shin DY (2009). Apoptosis induction of human lung carcinoma cells by Chan Su (*Venenum Bufonis*) through activation of caspases. *Journal of Acupuncture and Meridian Studies*.

[95] Wang J, Jin Y, Xu Z, Zheng Z, Wan S (2009). Involvement of caspase-3 activity and survivin downregulation in cinobufocini-induced apoptosis in A 549 cells. *Experimental Biology and Medicine*.

[96] Yu C-H, Kan S-F, Pu H-F, Chien EJ, Wang PS (2008). Apoptotic signaling in bufalin- and cinobufagin-treated androgen-dependent and -independent human prostate cancer cells. *Cancer Science*.

[97] Yeh J-Y, Huang WJ, Kan S-F, Wang PS (2003). Effects of bufalin and cinobufagin on the proliferation of androgen dependent and independent prostate cancer cells. *Prostate*.

[98] Gowda RM, Cohen RA, Khan IA (2003). Toad venom poisoning: resemblance to digoxin toxicity and therapeutic implications. *Heart*.

[99] Hu K, Zhu L, Liang H, Hu F, Feng J (2011). Improved antitumor efficacy and reduced toxicity of liposomes containing bufadienolides. *Archives of Pharmacal Research*.

[100] Li F, Weng Y, Wang L, He H, Yang J, Tang X (2010). The efficacy and safety of bufadienolides-loaded nanostructured lipid carriers. *International Journal of Pharmaceutics*.

[101] Ma H, Zhou J, Jiang J (2012). The novel antidote Bezoar Bovis prevents the cardiotoxicity of Toad (Bufo *bufo gargarizans* Canto) Venom in mice. *Experimental and Toxicologic Pathology*.

[102] Cao S, Cripps A, Wei MQ (2010). New strategies for cancer gene therapy: progress and opportunities. *Clinical and Experimental Pharmacology and Physiology*.

